# Blended Care in In-Patient Acute Psychiatric Care. The Example of a Group Training for Social Competences in Adults—A Pretest–Posttest Feasibility Study

**DOI:** 10.3390/ijerph18189433

**Published:** 2021-09-07

**Authors:** Eileen Bendig, Ann-Marie Küchler, Harald Baumeister, Thomas Becker

**Affiliations:** 1 Department of Clinical Psychology and Psychotherapy, Institute of Psychology and Education, Ulm University, 89081 Ulm, Germany; harald.baumeister@uni-ulm.de; 2 Department of Psychiatry II, Bezirkskrankenhaus Günzburg, Ulm University, 89312 Ulm, Germany; t.becker@uni-ulm.de

**Keywords:** blended care, psychiatry, social skills

## Abstract

Introduction: Deficits in social skills can be an important modulating factor in the development and progress of various mental disorders. However, limited resources in inpatient care often impede effective social skills training. This study investigates the feasibility of a blended group training for social skills (SST) in an inpatient psychiatric setting. Methods and Analysis: For this one-group pretest–posttest trial, inpatients with a diagnosed mental disorder were recruited. Participation in the blended SST lasted four weeks and took place within usual inpatient psychiatric care. The blended intervention comprised four face-to-face group sessions and three complementary online modules within four weeks. Assessments took place before (t1) and after (t2) the training. Feasibility outcomes (use, acceptance, satisfaction, implementability into usual psychiatric inpatient care) and effectiveness outcomes regarding social skills were assessed. Results: *N* = 15 participants were recruited. Most patients completed all questionnaires (93%) and all modules of the blended SST concept (60%). All participants (100%) would recommend the blended intervention to a friend. Regarding social skills, exploratory analyses revealed a non-significant medium-sized effect (Cohen’s d = 0.5 95%CI 0.3 to 1.25, *p* = 0.08). Discussion: This trial shows that a blended care SST is feasible for the use in acute psychiatric inpatient care setting. Although the wards were acute, with high turnover and change of inpatients, 60% of participants were treated per protocol over four weeks. Overall, the evidence on blended care concepts in psychiatric care settings is extremely poor to date. Hence, this trial should encourage intensified blended inpatient psychiatric care research.

## 1. Introduction

Social skills training can be an effective treatment in the field of many mental disorders such as social phobia, depression, or schizophrenia [1,2,3,4]. Although mental disorders usually have multi-causal origins, deficits in social skills have been found to be an important modulating factor not only for the development of mental disorders but also for their progress and consequences [5]. Therefore, the promotion of social skills is an established component of many psychosocial intervention approaches. Social skills training (SST) is regularly conceptualized as a systematic intervention that comprises behavioral techniques and strategies originally based on social learning theory [6]. Asserting one’s own rights, actively shaping relationships, or expressing feelings and needs are important components of psychosocial health [4,7]. The rationale for the use of SST in acute psychiatric care is based on evidence regarding its effectiveness.

Empirically, SST is well researched [6,7,8]. For years, researchers have been investigating the concept in the context of psychiatry and psychotherapy [4,9,10]. Across different mental disorders, studies suggest that strengthening individuals´ social skills can help to reduce symptoms [3,11,12]. One meta-analysis on randomized-controlled trials compared SST for people living with schizophrenia to active control groups or treatment-as-usual. In favor of the SST group, the authors found a weighted mean effect size of d = 1.20 for immediate content-mastery exams as well as d = 0.52 for an increase in performance-based measures of social and daily living skills and d = 0.52 for community functioning [3].

A promising way to enhance traditional inpatient services is blended therapy, in which face-to-face therapy is complemented by online modules [13,14]. Blended therapy makes it possible to outsource certain aspects of a treatment such as psychoeducation or regular exercises. These time-intensive elements can be worked on separately from a face-to-face setting. On the one hand, this frees therapeutic resources that can be used for face-to-face hands-on training (e.g., role plays). On the other hand, this can enable the inclusion of patients with different mental disorders, symptom severities, and cognitive functioning, as everyone can take his/her time to acquire knowledge, to repeat certain contents, and to practice in different frequencies.

The concept of blended therapy can come with advantages compared to online modules or face-to-face-sessions only. Integrating online modules to face-to-face settings can increase the treatment dosage without increasing treatment costs [14]. Online modules can help to foster the transfer of contents into everyday life and to support behavioral changes [14]. In comparison to stand-alone internet interventions, the patient acceptance can be higher [15]. In three recent studies, the required therapy time could be reduced by outsourcing certain elements to online modules without a loss in effectiveness [14].

Some studies indicate that blended group concepts could be a feasible approach. Schuster and colleagues [16] compared an eight-week computer- and multimedia-supported psychoeducational group intervention for adults with depressive symptoms to a control group within a randomized controlled trial (N = 46) [16]. The authors found the blended intervention concept to be feasible and acceptable. Additionally, the authors reported initial indications of its effectiveness with a large effect size of d = 0.87 (CI 0.26–1.46) for the reduction of depressive symptoms [16].

However, evidence for blended group therapy is scarce in general and even more in the context of acute inpatient psychiatric care, where to the authors’ knowledge, no study has been conducted so far. According to a review by Dülsen and colleagues [17] on digital interventions in adult mental healthcare settings, a similar situation presents itself for individual blended therapy in the inpatient setting. Only one RCT (N = 229) by Zwerenz and colleagues [18] investigated a blended therapy concept for people with depressive symptoms in a psychosomatic clinic and found small to medium effect sizes in comparison to an active control group (d = 0.44). Taken together, evidence for blended group training is extremely poor [17], but available study results are promising. To date, no study has investigated the effectiveness of a blended group SST in outpatient or inpatient samples. The focus of the present trial is initially on the feasibility of blended SST (use, acceptance, and satisfaction) and its potential effectiveness.

The research questions are as follows:

1. What is the extent of adherence, acceptance, and satisfaction?
2. Is there a change in social skills from pre- to post-measurement?
3. How can the concept be optimized for integration into inpatient psychiatry setting?

## 2. Materials and Methods

### 2.1. Study Design

The feasibility of a blended SST was evaluated within a quasi-experimental one-group pretest–posttest design. Patients could participate as part of inpatient treatment in the psychiatric care facility. The present trial was implemented in compliance with the Declaration of Helsinki and good scientific practice [19]. The trial is registered at the WHO International Clinical Trials Registry Platform via the German Clinical Studies Trial Register (DRKS): DRKS00022867 (date of registration: 27 August 2020). All procedures involved in the study are consistent with the generally accepted standards of ethical practice approved by the ethics committee of the University of Ulm (No. 190/20). The present study was conducted and is reported in accordance with the CONSORT 2010 guidelines for RCTs [20] and is in line with the SPIRIT Statement [21].

Routine care in the two participating wards comprised various therapeutic offers. This included a multimodal treatment concept (e.g., music therapy, art therapy, individual and group psychological offers, physical activity). Whereas one ward had a focus on younger adults with psychosis, both wards were acute psychiatric inpatient care units. Consequently, people with different mental disorders of varying severity were treated.

### 2.2. Eligibility Criteria

Participants needed to meet the following eligibility criteria: (a) fluent German language skills, (b) inpatient admission at Department of Psychiatry II of Ulm University at Bezirkskankenhaus (BKH) Günzburg, (c) age of 18 years or older, (d) diagnosed mental disorder according to the F-Chapter of the ICD-10 except F00, F02–F05 (dementia), and F71–F74 (intelligence reduction), (e) mild to severe functional limitations due to the mental disorder, HoNOS-D < 31).

### 2.3. Setting/Recruitment/Procedure

Recruitment took place from October to December 2020. This clinical study was conducted at the Department of Psychiatry II of Ulm University at the BHK Günzburg. Eligible patients were contacted by study personnel and received detailed study information following their informed consent.

Participants completed the baseline survey (paper–pencil) (t0) and took part in the four-week blended SST afterwards. After completion of the blended SST, participants received the post-measurement questionnaire (t1). At the end of each online session, participants gave formative feedback on the online modules within the Minddistrict^®^ platform.

### 2.4. Intervention

The blended SST was based on the evidence-based group training of social skills from Hinsch and Pfingsten [7]. It included four weekly face-to-face group sessions (duration 60 min). The face-to-face sessions were conducted in tandem by two psychotherapists in training (A-MK and EB). One psychologist had the role of the therapist and was responsible for guiding participants through the group SST protocol. The other psychologist was assigned the role of co-therapist and, besides serving as a partner for role-play demonstrations, also had the task to ensure therapist adherence to the group SST protocol. Between the face-to-face sessions, patients received one online module per week for preparation and follow-up of the contents of the face-to-face sessions (duration: approximately 15–30 min each). The brief duration of only four weeks was chosen deliberately due to the often short duration of stay in acute psychiatric inpatient care. Detailed information on intervention contents can be retrieved from Table 1.

Whilst the first session was conceptualized as introduction, the following face-to-face sessions focused on different types of social situations, respectively. The second one was on asserting one’s own right, the third was on relationships, and the fourth was on winning sympathy. After the introduction session, participants were encouraged to start with processing through the first of three online modules until the next face-to-face session. A detailed description of contents of the face-to-face sessions and online modules can be found in Table 1. Each online module consisted of psychoeducational input and exercises. Online modules were designed in a brief and interactive way, including examples, pictures, info boxes, and quizzes. Face-to-face sessions started with a repetition of the online module respectively, enriched with additional psychoeducational input. Subsequently, theoretical input was practiced in role plays. Situations were first played through by the therapists and afterwards by volunteering patients. After receiving initial feedback, the role play was repeated two times. The ratio of theoretical input and practical exercise in face-to-face appointments was about 15 to 45 min, representing a strong focus on practical exercises.

Online modules were offered on the Minddistrict^®^ platform. Minddistrict^®^ is a company specialized in the provision of internet interventions. Participants could access the platform 24 h using their personal tablet as provided by the hospital. All transferred data were secured on the basis of ISO27001 and the guidelines NEN7510. The intervention was carried out over a period of 4 weeks. Measurements were performed before and after the intervention.

### 2.5. Assessments and Outcome: Feasibility

Assessments took place before (t1) and after (t1) the blended SST in paper–pencil format. If patients unexpectedly terminated their stay at the hospital prior to completion, they were asked to fill out the post-treatment assessment. An overview of outcomes and measurements can be seen in Table 2.

#### 2.5.1. Adherence

Adherence was measured by recording the number of presence appointments and online modules completed by each individual patient. As the duration of stay in inpatient psychiatric inpatient care varies greatly and stays will sometimes end abruptly or unpredictably due to patients’ wishes or other reasons, we also documented reasons for discontinuation of the blended group SST (end of hospital stay vs. other reasons).

#### 2.5.2. Acceptance

A German translation of the “Attitude/affect toward using technology”-scale (ATT) [22] was used to measure patients’ acceptance of the therapeutic online material. Items of the scale are adapted from the original UTAUT (Unified Theory of Acceptance and Use of Technology) questionnaire by Venkatesh and colleagues [23]. The ATT consists of four items that range from 0 = “total disagreement” to = “strong agreement” on a 5-point Likert scale. Higher scores indicate higher acceptance, range: 0–20.

#### 2.5.3. Satisfaction

To assess patient satisfaction, the German revised short version ZUF-8 [24] of the Client Satisfaction Questionnaire (CSQ-8) [25] was used. This reliable [26] and short questionnaire measures satisfaction with the Blended Group SST. The CSQ-8 consists of eight items on a 4-point Likert scale with varying answers, resulting in a sum score. Higher scores indicate higher satisfaction. The CSQ-8 has demonstrated good psychometric properties with Cronbach’s α of 0.88 to 0.92 [27].

#### 2.5.4. Formative Feedback

To measure satisfaction with each of the individual online modules, patients answered six questions at the end of each online module on the Minddistrict^®^ platform, concerning their general liking of the module, its length, comprehensibility, and ideas for improvement.

#### 2.5.5. Social Insecurity

The assessment of social skills and social fears was measured with the Insecurity Questionnaire (U-Bogen-24) [28]. Participants rate 24 items on a 6-point Likert scale from 0 = “completely incorrect” to 5 = “completely correct”. The questionnaire consists of the four subscales fear of criticism, (e.g., “I am constantly afraid of saying or doing something wrong”), incapability of saying no (e.g., “I refrain from doing anything that could cause protest”), being able to make demands (e.g., “I can easily enforce my demands”), and fear of social contact (e.g., “It is embarrassing for me to ask a favor from friends”). Internal reliability has been found to be good with Cronbach´s α between 0.73 and 0.87 [28].

#### 2.5.6. Severity of Mental Disorder

Severity of mental illness was assessed using the clinician-rating instrument HoNOS-D [29]. The questionnaire covers four key areas of functioning: behavior (e.g., problematic alcohol or drug consumption), impairment (e.g., cognitive impairment), symptoms (e.g., depressed mood) and social functioning (e.g., relationship problems) on 12 items, each ranging from 0 (= no problem) to 4 (= severe problem). The total score representing overall severity ranges between 0 and 48 with higher scores indicating higher severity. Regarding test–retest reliability, intraclass coefficients have been found to be between 0.72 and 0.91 [30].

## 3. Sample Size Estimation

As this feasibility study may help with the development of the intervention and outcome measures, the target of this trial was to recruit up to 32 participants overall. In accordance with the extended version of the CONSORT Statement for feasibility trials [31], investigators decided to proceed with the recruitment of participants until enough information was gathered to ensure feasibility for the investigation within a potential future definitive trial.

Due to the COVID-19 pandemic, wards were temporarily closed, and the implementation of group therapy was only possible to a limited extent. Consequently, the start of the trial had to be postponed multiple times, resulting in a shorter time of data collection than expected. However, research questions could be addressed to a satisfactory extent with a sample size of 15 participants.

## 4. Statistical Analyses

In order to assess the feasibility of the present study, the statistical analysis was carried out on a descriptive level. Sample characteristics were described in absolute and relative frequencies. For metric variables, descriptive statistics with mean values (M) and the standard deviation (SD) were calculated. On an exploratory level, a *t*-test for paired samples was used to quantify descriptive differences in the mean insecurity pre- and post-treatment. Since individual items of the insecurity questionnaire were missing (in total 1%) [32], these were replaced by the mean value across all participants of the respective item to enable calculation of sum- and sub-scores. One participant left the hospital hastily and without consultation with the treatment staff. The participant could not be reached afterwards for completion of T1 assessment and therefore was excluded from further analyses. However, to comply with principles of intention-to-treat (ITT) analyses, he was included in all other analyses.

## 5. Results

### 5.1. Baseline Participant Characteristics

Within the one-month recruitment period, N = 15 individuals met the inclusion criteria and gave informed consent. The mean age of participants was M = 34.53 (SD = 11.19) ranging from 19 to 58 years. The gender ratio was balanced (*n* = 7 female, *n* = 8 male). Baseline participant characteristics are presented in Table 3. Baseline HoNOS-score was M = 12.50 (SD = 2.53), range 10–20. Questionnaires descriptive statistics for pre- and post-measurement are presented in Table 4.

### 5.2. Feasibility

With regard to study adherence, most patients (93%) completed pre- and post-measurements. One patient (7%) prematurely left the hospital and did not complete post-measurement questionnaires. With regard to intervention adherence, in total, 60% (*n* = 9) of participants adhered to the Blended SST concept (processed through all presence and online modules). Regarding face-to-face adherence, one participant processed through two face-to-face sessions, six patients processed through three face-to-face sessions, and eight patients processed through all four face-to-face sessions. Non-adherence with face-to-face sessions was due to early departure from the hospital. Four other patients did not adhere to all of the online modules, as they left the hospital prior to processing through the last online module. Thereby, *n* = 2 (13%) completed one, *n* = 2 (13%) completed two, and *n* = 11 (74%) completed all three of the additional online modules. Average patients’ acceptance (ATT) was M = 12.31 (SD = 3.50). On a descriptive level, patients’ acceptance slightly decreased from T0 to T1 (T1: M = 11.93, SD = 3.77) (Table 5).

The average satisfaction (CSQ-8) with the Blended SST at T1 was M = 26.00 (SD = 4.52). All participants (100%) would probably/definitively recommend the blended intervention to a friend. Most participants (79%) rated the quality of the intervention as good or excellent. Formative feedback indicated that participants rated the online modules on average with 8 out of 10 points (L1: M = 7.64, SD = 2.55; L2: M = 7.98, SD = 1.80; L3: M = 8.42, SD = 1.10) at a low average processing time of 13 min, ranging from 4 to 40 min (L1: M = 12.54, SD = 9.33; L2: M = 9.33, SD = 7.05; L3: M = 15.91, SD = 10.32). Most participants (66%) rated the time spend with the online modules as just right (L1: 86.7%, L2: 69.2%, L3: 72.2%) and were able to understand the contents easily (L1: 93.3%, L2: 84.6%, L3: 81.8%). The most helpful components were case examples and exercises as well as techniques and information about the different topics. Suggestions for improvement mainly comprised more exercises, examples, and the wish for more and responsive content.

### 5.3. Potential Effectiveness

To explore potential change in social skills from pre- to post-measurement, analyses indicated that the mean insecurity scores decreased from T0 to T1 from M = 55.94 (SD = 17.17) to M = 50.79 (SD = 16.01). This difference, 5.14, 95% CI (−0.70 to 10.99), was not significant t(13) = 1.90, *p* = 0.08 and represents a medium-sized pre–post effect of d = 0.5 95% CI (0.3 to 1.25).

## 6. Discussion

Blended therapy approaches, meaning the combination of face-to-face therapy with digital elements, could contribute substantially to a better provision of psychotherapy in inpatient care. However, evidence is scarce and even more so in acute psychiatric care. Research designs are needed that allow investigating the feasibility of such approaches within the inpatient setting. Therefore, in this quasi-experimental pretest–posttest feasibility study, we evaluated the feasibility of a blended social skills training (SST) in the setting of an acute psychiatric inpatient clinic. The blended SST was found to be feasible with regard to acceptance measured via patients’ attitudes toward complementary online modules, intervention adherence regarding both face-to-face sessions and online modules, and intervention satisfaction with the blended SST as a whole. Additionally, we found preliminary evidence for a potential effectiveness of blended SST, however, with non-significant pre–post effects in this study, which has not been powered for effectiveness testing.

Regarding sample characteristics, participants’ average severity of mental disorder (HoNOS) was M = 12.60. Although there is little research concerning sensible cut-offs for various severity levels, this finding matches research from Prowse and Coombs [33] that found a cut-off of 12 as being indicative of the need for intensive psychiatric care. Moreover, results show that the intervention was feasible for patients with this severity level. Furthermore, we found the gender ratio to be balanced with participants across the young-to-middle-aged adult lifespan (19–58). Consequently, our sample, although relatively small, seemed to have mirrored the diverse patient clientele in general acute psychiatric inpatient hospitals and suggests the feasibility of the blended SST in this population.

Concerning evaluation of intervention adherence, the duration of stay of patients in acute psychiatric inpatient hospitals is less predictable than in the outpatient setting due to various reasons such as early (self-)release. Additionally, patients often present more severe and comorbid mental health problems compared to outpatient psychiatric and psychotherapeutic care [34]. As a result, implementing psychotherapeutic interventions in this setting can be challenging. Nevertheless, we observed that 60% of patients attended all face-to-face-sessions and completed all three online modules over the blended intervention period of four weeks. This indicates that the implementation of a blended care concept over several weeks appears to be feasible in acute psychiatric inpatient settings. Specifically, 74% of patients completed all three online modules on their own between the face-to-face sessions. This is promising, especially since adherence problems are repeatedly reported in stand-alone online interventions [35,36]. Acceptance of online components within the blended SST can also be confirmed as patients generally showed a positive attitude toward the integration of online modules into group SST.

Finally, participants were satisfied with the blended SST concept as a whole, exceeding the cut-off of 24.5 suggested by Kriz and colleagues [27] for measuring intervention satisfaction in psychosomatic patients. With regard to online module contents and length, module contents were generally rated as easily comprehensible, and two-thirds of participants were satisfied with the average processing time (M = 13 min), whereas the rest rated the modules as too short. These results show that module content and length are feasible for a future definitive trial in an acute psychiatric sample; however, further development of module provision more tailored to patients’ needs and preferences might further improve interventions’ persuasiveness and thus potential effectiveness [37].

As evidence concerning blended group therapy is very scarce and this is the first trial to investigate the feasibility of blended group SST, comparison of results to existing studies is only possible to a limited extent. Our results of feasibility are in line with results from an RCT by Schuster and colleagues [16] who found an 8-week computer- and multimedia-supported psychoeducational group intervention for adults with depressive symptoms to be acceptable and feasible. Additionally, our results extend existing evidence to the population of patients of acute psychiatric inpatient hospitals and the treatment approach of social skills training.

With regard to insecurity as an indicator of potential effectiveness, the findings highlight a decrease in average insecurity from pre- to post-treatment in the range of a medium effect size. However, this difference remained non-significant. It is unlikely to detect significant changes in such a small sample. A posteriori power analyses (two-tailed *t*-test paired samples, G*power, 3.1.9.7.) showed that a sample size of N = 44 would have been necessary to detect an effect of 0.5 with a power of 0.9. Nonetheless, results suggest insecurity to be potentially improvable by the blended SST in the range of a medium-sized pre–post-treatment effect and therefore suitable as a primary outcome measure in a potential confirmatory RCT.

### 6.1. Blended SST in Acute Inpatient Psychiatry: Challenges and Opportunities

First and foremost, this feasibility trial revealed the complex structures of acute psychiatric inpatient care that have to be considered when planning a definitive randomized controlled trial for the evaluation of a blended care group concept in this setting. Various stakeholders should be included early in the planning and conduct of the study, such as management, senior physicians, psychotherapeutics, and nursing staff. Data protection requirements are specifically strict within the hospital setting, which is all the more relevant for treatment concepts that include digital elements. Consequently, aspects of data security as well as technical aspects such as the availability of Wi-Fi and tablets have to be considered early on. On the one hand, patients’ stays are often brief, whereas on the other hand, patients’ symptomatology is often much more severe than in outpatient care. Nevertheless, disorder severity level varies greatly, and acute psychiatric hospital wards often treat patients with a wide variety of disorders. Treatment concepts that are to be implemented in this setting need to account for these circumstances and should be as inclusive as possible. Thereby, findings regarding the comparable effectiveness of transdiagnostic versus disorder-specific Internet-based anxiety interventions [38] suggest that transdiagnostic and competence-focused offers might be helpful as well and thus potentially more useful in a setting with diverse patients, disorders, and severity levels.

Regarding tailoring, contents of the Online SST modules were kept brief on purpose to enable access for patients with more severe symptom levels. However, modules were rated too short by one-third of participants, and formative feedback revealed that adding more content (e.g., examples, exercises) was the most frequently expressed suggestion for improvement. To address the varying needs and symptom levels of individual patients, conditional content could be added to the modules (e.g., further information, exercises) as addition to core elements. Then, patients could decide while working through the modules whether they want to be presented with further content or not. By tailoring online modules in this way, satisfaction levels could be optimized [39].

### 6.2. Limitations

Several limitations need to be taken into consideration.

First, the trial took place in times of the COVID-19 pandemic, which brought various challenges such as the temporary closure of wards and ban of group treatment formats. The trial start had to be postponed multiple times. Consequently, a small sample size of 15 patients was reached. This limits the validity of statistical results. However, the primary research questions of feasibility could be answered.

Second, this trial did not have a control group, as the main research question was to determine whether the blended treatment concept is feasible in the setting of an acute psychiatric hospital. However, conclusions on effectiveness cannot be drawn because of different factors. On the one hand, the small sample size made it unlikely that the statistical comparison of means would become significant. On the other hand, descriptive changes concerning the outcome variable insecurity might have been confounded by other treatment elements of routine care. In order to be able to make statements about the effectiveness, full-scale definitive trials are required. This should include active control groups, such as pure face-to-face group SST, to examine the non-inferiority of blended care [13].

Further, the generalizability of results is limited in two ways. First, as we decided to include patients of most diagnostic categories, we cannot draw any valid conclusions concerning differences in feasibility between different mental disorder diagnoses. However, we made this decision deliberately to mirror the inpatient acute psychiatric care population, therefore increasing the external validity of results. Second, for a cut-off of HoNOS-D, < 31 was defined to exclude patients with an extremely high severity of mental disorder that would prevent them from actively taking part in the blended SST. Participants had a mean HoNOS-D of 12.5 in our sample, with a range from 10 to 20. Therefore, the results cannot be generalized to people with higher severity of mental disorder.

Finally, three of the authors of this trial were involved in treatment design, conduct, and evaluation, which might have heightened the risk of allegiance bias [40].

### 6.3. Conclusions

To the best of our knowledge, this study is the first investigating a blended social skills group training in an inpatient acute psychiatric care setting. This study particularly confirmed the feasibility of such an intervention in this type of setting. Results might encourage effectiveness studies in this underrepresented area of research. In the long term, this will contribute to the improvement of psychiatric patients’ provision with psychotherapeutic group therapy by providing important information on the implementation of blended care concepts into routine acute psychiatric inpatient care.

## Figures and Tables

**Table 1:** Content of Blended Group SST.

Week	Presence Appointment	Online Module
1st week	Appointment 1: Introduction Input: what are social skills, why are they relevant, types of situations social skills are needed, discrimination training Practical training: giving and receiving compliments	
		Online module 1: “Asserting one’s right” Repetition: What are social skills? Input: the power of thinking, when to assert one’s right Exercise: which social competencies do you wish to work on, negative vs. helpful thinking, how to defend your booked seat in the train
2nd week	Appointment 2: “Asserting one’s right” Repetition: summary online module 1 Input: explanatory model on the consequences of positive vs. negative self-verbalization on feelings and behavior, development of instructions for self-confident behavior in “Asserting one’s right”—situations Practical training: role play Homework: in vivo practice of “Asserting one’s right”-situations	
		Online module 2: “Relationships” Input: differences between “Asserting one’s right” and “Conflicts in relationships”, perceiving one’s feelings and thoughts, disclosing yourself to others, listening to others Exercise: Perceiving one’s own body, thoughts, and feelings
3rd week	Appointment 3: “Relationships” Repetition: summary online module 2 Input: development of instructions for self-confident behavior in “Relationship”—situations Practical training: role play homework: practicing listening to and communicating feelings and needs	
		Online module 3: “Winning sympathy” Input: how to win someone over, the power of thinking, reinforcement strategies, how to conduct a conversation Exercise: helpful thoughts and behaviors, open and closed questions
4th week	Appointment 4: “Winning sympathy” Repetition: summary online module 3 Input: development of instructions for self-confident behavior in “Winning sympathy” situations Practical training: role play Conclusion: comparison of self-confident behavior in different situations, individual take-home-message	

Note. SST = group training of social skills.

**Table 2:** Outcomes and assessment points.

Variables	Measurement	t0	t1
Sociodemographic variables	SRQ	x	
Severity of mental disorder	HoNOS-D	x	
Diagnosis	Patient file	x	
Social insecurity	Insecurity Questionnaire	x	x
Adherence	Participation in classroom sessions and processing of online sessions		x
Acceptance	ATT	x	x
Satisfaction	CSQ-8		x
Feedback on online sessions	Open and closed questions at the end of the sessions		

Note. ATT = Attitudes Toward Technology, German Version; HoNOS-D = Health of the Nation Outcome Scales German Version; SRQ = Self-Report Questionnaire; CSQ-8 = Client Satisfaction Questionnaire.

**Table 3:** Sample characteristics.

	n (%)
Sex	
Female	7 (47)
Male	8 (53)
ICD-10 diagnosis	
F00–F09 Organic, including symptomatic, mental disorders	2
F10–F19 Mental and behavioral disorders due to psychoactive	2
substance use	
F20–F29 Schizophrenia, schizotypal, and delusional disorders	3
F30–F39 Mood (affective) disorders	6
F40–F48 Neurotic, stress-related, and somatoform disorders	2

**Table 4:** Questionnaires descriptive statistics at their points of measurement.

	Points of Measurement	
	T0	T1
	M (SD)	M (SD)
HoNOS-D	12.50 (2.53)	
U-Bogen-24		
fear of criticism	12.91 (8.51)	11.77 (6.68)
incapability of saying no	14.64 (2.98)	16.21 (2.26)
being unable to make demands	16.75 (4.16)	14.31 (6.02)
fear of social contact	11.64 (7.01)	8.50 (4.70)
total scale	55.94 (17.17)	50.79 (16.01)
ATT	12.31 (3.50)	11.93 (3.77)
CSQ-8		26.00 (4.52)

Note. HoNOS-D = Severity of mental illness; U-Bogen-24 = Insecurity Questionnaire; ATT = Attitudes Toward Technology Scale; CSQ-8 = Client Satisfaction Questionnaire; T0 = pre-measurement; T1 = post-measurement.

**Table 5:** Acceptance of digital treatment elements.

	Points of Measurement	
	T0	T1
	N(%)	N(%)
ATT items “agree or strongly agree”		
The online modules make group training more interesting	12 (71.50)	8 (61.60)
Using the online modules is a good idea	12 (85.70)	9 (69.20)
I think working with the online modules is fun	9 (64.30)	8 (61.60)
I think I will enjoy working with the online modules	9 (64.30)	8 (61.60)

Note. ATT = Attitudes Toward Technology; T0 = pre-measurement; T1 = post-measurement.

## Data Availability

Data will be made available upon reasonable request.

## Trial Registration

DRKS-ID: DRKS00022867.
